# Cholic acid–quinoxaline (2/1)

**DOI:** 10.1107/S1600536808015067

**Published:** 2008-05-21

**Authors:** Barbara Wicher, Maria Gdaniec

**Affiliations:** a Faculty of Chemistry, Adam Mickiewicz University, 60-780 Poznań, Poland

## Abstract

In the title inclusion compound, 2C_24_H_40_O_5_·C_8_H_6_N_2_, the unit cell contains two mol­ecules of cholic acid (3α,7α,12α-trihydr­oxy-5β-cholan-24-oic acid) and one mol­ecule of quinoxaline which implies disorder of the quinoxaline in the space group *P*2_1_. The amphiphilic mol­ecules of cholic acid assemble, in an anti­parallel arrangement, *via* O—H⋯O hydrogen bonds, into typical corrugated host bilayers which are lipophilic on the outside and lipophobic on the inside. The host framework belongs to the so called α-*trans* subtype. The quinoxaline mol­ecules are accommodated in lipophilic channels formed between neighboring bilayers with only van der Waals inter­actions between host and guest. There is a crystallographic twofold screw axis directed along an empty channel in the host framework; however, neighboring guests in any one channel are related by a unit-cell translation along the *b* axis. Thus, the overall structure is a 1:1 superposition of two such channels related by the crystallographic twofold screw axis.

## Related literature

For structural information on cholic acid inclusion compounds, see: Miyata & Sada (1996 ▶); Nakano *et al.* (2001 ▶, 2006 ▶). 
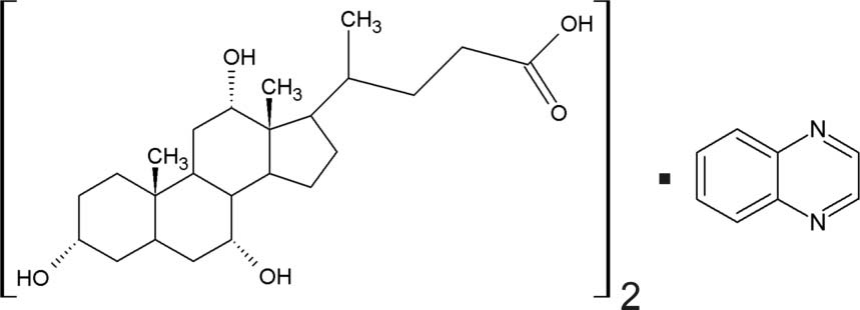


## Experimental

### 

#### Crystal data

2C_24_H_40_O_5_·C_8_H_6_N_2_
*M* *_r_* = 947.27
Monoclinic, 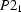

*a* = 12.2799 (5) Å
*b* = 7.8968 (3) Å
*c* = 14.2831 (5) Å
β = 104.653 (4)°
*V* = 1340.01 (9) Å^3^
*Z* = 1
Mo *K*α radiation
μ = 0.08 mm^−1^
*T* = 130 (2) K
0.6 × 0.2 × 0.09 mm

#### Data collection

Kuma KM-4-CCD κ-geometry diffractometer
Absorption correction: multi-scan (*SCALE3 ABSPACK* scaling algorithm; Oxford Diffraction, 2007 ▶) *T*_min_ = 0.783, *T*_max_ = 1.000 (expected range = 0.777–0.993)
9593 measured reflections
2929 independent reflections
2548 reflections with *I* > 2σ(*I*)
*R*_int_ = 0.016

#### Refinement

*R*[*F* ^2^ > 2σ(*F* ^2^)] = 0.035
*wR*(*F* ^2^) = 0.102
*S* = 1.07
2929 reflections
353 parameters
1 restraint
H-atom parameters constrained
Δρ_max_ = 0.23 e Å^−3^
Δρ_min_ = −0.17 e Å^−3^

### 

Data collection: *CrysAlis CCD* (Oxford Diffraction, 2007 ▶); cell refinement: *CrysAlis RED* (Oxford Diffraction, 2007 ▶); data reduction: *CrysAlis RED*; program(s) used to solve structure: *SHELXS97* (Sheldrick, 2008 ▶); program(s) used to refine structure: *SHELXL97* (Sheldrick, 2008 ▶); molecular graphics: *ORTEP-3 for Windows* (Farrugia, 1997 ▶) and *Mercury* (Macrae *et al.*, 2006 ▶); software used to prepare material for publication: *SHELXL97*.

## Supplementary Material

Crystal structure: contains datablocks global, I. DOI: 10.1107/S1600536808015067/fl2200sup1.cif

Structure factors: contains datablocks I. DOI: 10.1107/S1600536808015067/fl2200Isup2.hkl

Additional supplementary materials:  crystallographic information; 3D view; checkCIF report

## Figures and Tables

**Table 1:** Hydrogen-bond geometry (Å, °)

*D*—H⋯*A*	*D*—H	H⋯*A*	*D*⋯*A*	*D*—H⋯*A*
O1—H1*O*⋯O3^i^	0.82	2.03	2.815 (2)	161
O2—H2*O*⋯O1^i^	0.82	1.86	2.648 (2)	160
O3—H3*O*⋯O4^ii^	0.82	2.03	2.834 (2)	167
O5—H5*O*⋯O2^ii^	0.82	1.85	2.656 (2)	170

Symmetry codes: (i) 
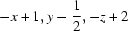
; (ii) 
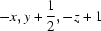
.

**Fig. 1.**
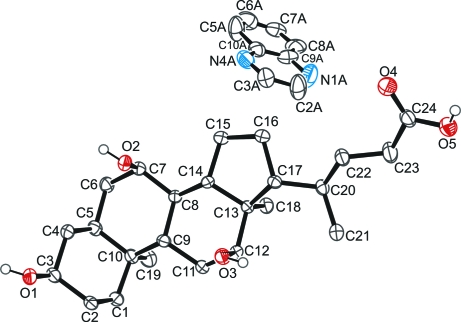
: The molecular structure of the title compound with displacement ellipsoids shown at the 50% probability level. H atoms bound to C atoms are omitted for clarity.

**Fig. 2.**
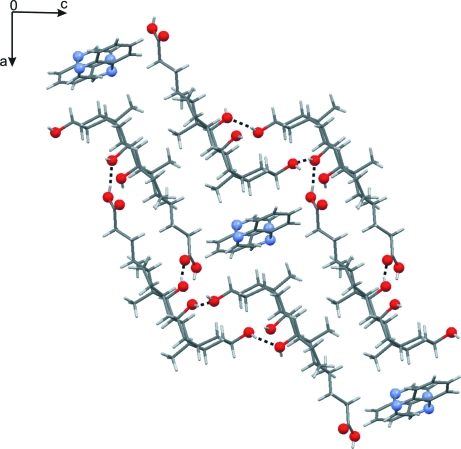
: Crystal packing viewed down the b axis. Hydrogen bonds are shown with dashed lines.

**Fig. 3.**
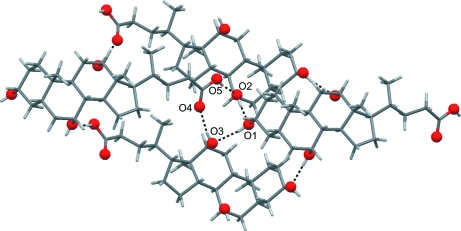
: Cyclic motif of O—H···O hydrogen bonds generating two-dimensional network of the host molecules.

**Fig. 4.**
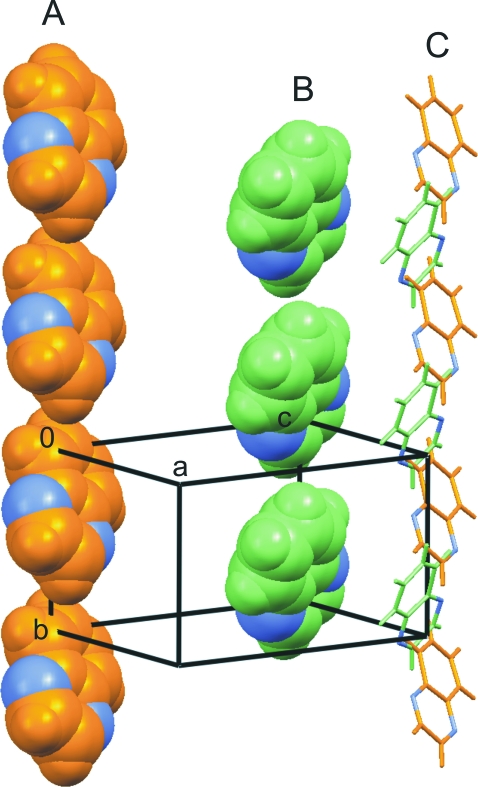
Arrangement of the guest molecules within the channels. In the channels A and B the molecules are related by translation operation. The channel C shows superposition of the channels A and B (related by the twofold screw axis), as seen from the electron-density maps.

## supplementary crystallographic information

### Comment

Cholic acid forms inclusion compounds with a large variety of guest molecules (Miyata & Sada, 1996). The host framework is strongly dependent on the guest but, in most cases, it is constructed from cholic acid bilayers which are lipophilic on the outside and lipophobic on the inside (Nakano *et al.*, 2001, 2006). The type of the host framework and the host:guest ratio are strongly dependent on the volume and shape of the guest  (Nakano *et al.*, 2001). In the case of bilayers with an antiparallel arrangement of host molecules, four framework subtypes  are generally recognized: α-*gauche*, α-*trans*, β-*gauche* and β-*trans* (Miyata & Sada, 1996) based on the conformation of the steroidal side chain (*gauche*/*trans*) and the stacking mode of the bilayers (α/β). Among numerous guest molecules that have cocrystallized with cholic acid no larger arenes or aromatic azaheterocycles have been reported. This is probably due to the problems with accommodating large molecules of fixed geometry within corrugated host channels. Quinoxaline easily cocrystallized with cholic acid, because as a low melting solid it could be used for cocrystallization without the need for any additional solvent.

In (I) ( Fig. 1), the  host molecules are arranged in typical antiparallel bilayers and the framework can be classified as α-*trans* (Fig. 2). Four molecules of the host generate a cyclic motif of O—H···O hydrogen bonds (Fig. 3, Table 1) that assembles molecules into a two-dimensional polymeric structure (host bilayer). The hydrogen bonds are not completely buried on the inside of the bilayer as they partially line the grooves on the corrugated bilayer surface. The quinoxaline molecules are accommodated in lipophilic channels formed between neighboring bilayers and there are only van der Waals interactions between host and guest. The unit cell contains two molecules of the bile acid and one molecule of quinoxaline. In *P*2_1 _this implies disorder of the guest and this is the case for (I): the crystallographic symmetry of the empty channel is higher than the symmetry of the guest arrangement within the channel. Neighbouring guests are related by translation along *b*  [7.8968 (3) Å] and not by the crystallographic 2_1_ axis operating along the channel (Fig. 4). There is no long-distance order in the channels  because no reflections in addition to the Bragg reflections were detected. Thus, the model of the crystal structure of the title compound reveals superposition of two channels related by the crystallographic twofold screw axis ( Fig. 4).

### Experimental

The title compound was obtained by dissolving cholic acid (0.1 g, 0.24 mmol) in melted quinoxaline (0.7 g, 5.38 mmol) and evaporation of the excess of quinoxaline at 60°C for two days. The resulting colorless plates were stable in air.

### Refinement

In the absence of significant anomalous scattering effects, Friedel pairs were averaged. The absolute configuration of cholic acid was assigned from the known configuration of the starting material. All H atoms were located in electron-density difference maps. For refinement all H atoms were placed at calculated positions, with C—H = 0.96–0.98 Å and O—H = 0.82 Å, and were refined as riding on their carrier atoms with *U*_ĩso_(H) = 1.2*U*_eq_(C, O). No restraints were imposed on geometry of the disordered quinoxaline molecules (occupancy factor 1/2).

### Crystal data

**Table:**

2C_24_H_40_O_5_·C_8_H_6_N_2_	*F*_000_ = 516
*M**_r_* = 947.27	*D*_x_ = 1.174 Mg m^−^^3^
Monoclinic, *P*2_1_	Mo *K*α radiation λ = 0.71073 Å
Hall symbol: P 2yb	Cell parameters from 6598 reflections
*a* = 12.2799 (5) Å	θ = 2.0–27.7º
*b* = 7.8968 (3) Å	µ = 0.08 mm^−^^1^
*c* = 14.2831 (5) Å	*T* = 130 (2) K
β = 104.653 (4)º	Plate, colorless
*V* = 1340.01 (9) Å^3^	0.6 × 0.2 × 0.09 mm
*Z* = 1	

### Data collection

**Table:**

Kuma KM-4-CCD κ-geometry diffractometer	2929 independent reflections
Radiation source: fine-focus sealed tube	2548 reflections with *I* > 2σ(*I*)
Monochromator: graphite	*R*_int_ = 0.016
*T* = 130(2) K	θ_max_ = 26.4º
ω scans	θ_min_ = 4.3º
Absorption correction: multi-scan(SCALE3 ABSPACK scaling algorithm; Oxford Diffraction, 2007)	*h* = −14→15
*T*_min_ = 0.783, *T*_max_ = 1.000	*k* = −7→9
9593 measured reflections	*l* = −17→17

### Refinement

**Table:**

Refinement on *F*^2^	Secondary atom site location: difference Fourier map
Least-squares matrix: full	Hydrogen site location: inferred from neighbouring sites
*R*[*F*^2^ > 2σ(*F*^2^)] = 0.035	H-atom parameters constrained
*wR*(*F*^2^) = 0.102	*w* = 1/[σ^2^(*F*_o_^2^) + (0.0726*P*)^2^] where *P* = (*F*_o_^2^ + 2*F*_c_^2^)/3
*S* = 1.07	(Δ/σ)_max_ = 0.004
2929 reflections	Δρ_max_ = 0.23 e Å^−^^3^
353 parameters	Δρ_min_ = −0.17 e Å^−^^3^
1 restraint	Extinction correction: none
Primary atom site location: structure-invariant direct methods	

### Special details

**Table:**

Geometry. All e.s.d.'s (except the e.s.d. in the dihedral angle between two l.s. planes) are estimated using the full covariance matrix. The cell e.s.d.'s are taken into account individually in the estimation of e.s.d.'s in distances, angles and torsion angles; correlations between e.s.d.'s in cell parameters are only used when they are defined by crystal symmetry. An approximate (isotropic) treatment of cell e.s.d.'s is used for estimating e.s.d.'s involving l.s. planes.
Refinement. Refinement of *F*^2^ against ALL reflections. The weighted *R*-factor *wR* and goodness of fit *S* are based on *F*^2^, conventional *R*-factors *R* are based on *F*, with *F* set to zero for negative *F*^2^. The threshold expression of *F*^2^ > σ(*F*^2^) is used only for calculating *R*-factors(gt) *etc*. and is not relevant to the choice of reflections for refinement. *R*-factors based on *F*^2^ are statistically about twice as large as those based on *F*, and *R*- factors based on ALL data will be even larger.

### Fractional atomic coordinates and isotropic or equivalent isotropic displacement parameters (Å^2^)

**Table:**

	*x*	*y*	*z*	*U*_iso_*/*U*_eq_	Occ. (<1)
O1	0.59222 (14)	0.4911 (2)	1.10590 (10)	0.0314 (4)	
H1O	0.6242	0.4236	1.1475	0.038*	
O2	0.42660 (12)	0.2628 (2)	0.79071 (10)	0.0258 (3)	
H2O	0.4339	0.1702	0.8176	0.031*	
O3	0.32033 (13)	0.8075 (2)	0.72265 (10)	0.0274 (3)	
H3O	0.2725	0.8813	0.7056	0.033*	
O4	−0.16441 (14)	0.5786 (2)	0.30332 (13)	0.0426 (5)	
O5	−0.20929 (13)	0.8331 (2)	0.23696 (11)	0.0323 (4)	
H5O	−0.2738	0.8000	0.2320	0.039*	
C1	0.69475 (18)	0.7038 (3)	0.90818 (16)	0.0274 (5)	
H1A	0.7753	0.6938	0.9355	0.033*	
H1B	0.6802	0.8167	0.8812	0.033*	
C2	0.63568 (19)	0.6848 (3)	0.98942 (15)	0.0255 (5)	
H2A	0.6637	0.7698	1.0386	0.031*	
H2B	0.5555	0.7026	0.9640	0.031*	
C3	0.65639 (19)	0.5100 (3)	1.03418 (15)	0.0275 (5)	
H3A	0.7367	0.4957	1.0651	0.033*	
C4	0.61870 (19)	0.3768 (3)	0.95714 (15)	0.0260 (5)	
H4A	0.5376	0.3832	0.9329	0.031*	
H4B	0.6368	0.2660	0.9863	0.031*	
C5	0.67282 (18)	0.3938 (3)	0.87164 (15)	0.0254 (5)	
H5A	0.7538	0.3747	0.8972	0.030*	
C6	0.62925 (18)	0.2534 (3)	0.79734 (15)	0.0270 (5)	
H6A	0.6791	0.2449	0.7546	0.032*	
H6B	0.6324	0.1465	0.8313	0.032*	
C7	0.50925 (17)	0.2804 (3)	0.73578 (14)	0.0236 (4)	
H7A	0.4935	0.1963	0.6836	0.028*	
C8	0.49507 (17)	0.4575 (3)	0.69055 (14)	0.0208 (4)	
H8A	0.5416	0.4633	0.6441	0.025*	
C9	0.53446 (17)	0.5997 (3)	0.76600 (14)	0.0209 (4)	
H9A	0.4872	0.5932	0.8119	0.025*	
C10	0.65901 (17)	0.5734 (3)	0.82549 (15)	0.0235 (5)	
C11	0.51251 (17)	0.7736 (3)	0.71581 (14)	0.0236 (4)	
H11A	0.5315	0.8613	0.7648	0.028*	
H11B	0.5625	0.7862	0.6734	0.028*	
C12	0.39105 (16)	0.8019 (3)	0.65640 (14)	0.0221 (4)	
H12A	0.3865	0.9109	0.6228	0.027*	
C13	0.35326 (16)	0.6593 (3)	0.58027 (14)	0.0193 (4)	
C14	0.37382 (17)	0.4900 (3)	0.63560 (14)	0.0192 (4)	
H14A	0.3301	0.4950	0.6842	0.023*	
C15	0.31647 (18)	0.3578 (3)	0.56138 (15)	0.0245 (5)	
H15A	0.2953	0.2588	0.5930	0.029*	
H15B	0.3654	0.3231	0.5211	0.029*	
C16	0.21215 (18)	0.4509 (3)	0.50161 (15)	0.0251 (5)	
H16A	0.1445	0.4049	0.5151	0.030*	
H16B	0.2067	0.4381	0.4330	0.030*	
C17	0.22533 (16)	0.6422 (3)	0.53071 (14)	0.0194 (4)	
H17A	0.1849	0.6601	0.5809	0.023*	
C18	0.41975 (17)	0.6735 (3)	0.50303 (15)	0.0249 (5)	
H18A	0.4973	0.6463	0.5313	0.030*	
H18B	0.4142	0.7870	0.4782	0.030*	
H18C	0.3893	0.5960	0.4512	0.030*	
C19	0.74162 (19)	0.5929 (4)	0.76107 (17)	0.0334 (5)	
H19A	0.8157	0.5596	0.7968	0.040*	
H19B	0.7430	0.7090	0.7414	0.040*	
H19C	0.7177	0.5224	0.7049	0.040*	
C20	0.17040 (17)	0.7576 (3)	0.44491 (13)	0.0224 (4)	
H20A	0.2059	0.7344	0.3920	0.027*	
C21	0.18378 (19)	0.9459 (3)	0.46903 (16)	0.0287 (5)	
H21A	0.2621	0.9756	0.4842	0.034*	
H21B	0.1541	0.9698	0.5237	0.034*	
H21C	0.1435	1.0109	0.4144	0.034*	
C22	0.04541 (17)	0.7089 (3)	0.41068 (15)	0.0263 (5)	
H22A	0.0403	0.5866	0.4047	0.032*	
H22B	0.0085	0.7418	0.4604	0.032*	
C23	−0.01830 (19)	0.7862 (4)	0.31591 (17)	0.0382 (6)	
H23A	−0.0199	0.9083	0.3227	0.046*	
H23B	0.0205	0.7603	0.2663	0.046*	
C24	−0.1376 (2)	0.7201 (3)	0.28483 (15)	0.0300 (5)	
N1A	−0.0432 (7)	1.0289 (10)	0.9084 (5)	0.083 (2)	0.50
C2A	−0.0825 (14)	1.0781 (18)	0.9829 (10)	0.080 (2)	0.50
H2Q	−0.1006	1.1947	0.9906	0.095*	0.50
C3A	−0.0749 (6)	0.9642 (11)	1.0630 (5)	0.0547 (16)	0.50
H3Q	−0.1167	0.9897	1.1097	0.066*	0.50
N4A	−0.0260 (5)	0.8179 (8)	1.0700 (4)	0.0596 (14)	0.50
C5A	0.0703 (14)	0.6025 (17)	0.9949 (11)	0.080 (2)	0.50
H5Q	0.0778	0.5261	1.0485	0.095*	0.50
C6A	0.1128 (8)	0.5634 (13)	0.9238 (6)	0.076 (2)	0.50
H6Q	0.1481	0.4559	0.9204	0.091*	0.50
C7A	0.1092 (5)	0.6676 (10)	0.8484 (5)	0.0579 (17)	0.50
H7Q	0.1446	0.6350	0.7985	0.070*	0.50
C8A	0.0569 (6)	0.8161 (11)	0.8407 (5)	0.066 (2)	0.50
H8Q	0.0534	0.8864	0.7852	0.079*	0.50
C9A	0.0065 (5)	0.8732 (9)	0.9145 (5)	0.0532 (16)	0.50
C10A	0.0145 (4)	0.7716 (9)	0.9960 (5)	0.0493 (14)	0.50

### Atomic displacement parameters (Å^2^)

**Table:**

	*U*^11^	*U*^22^	*U*^33^	*U*^12^	*U*^13^	*U*^23^
O1	0.0475 (10)	0.0242 (9)	0.0197 (7)	0.0043 (7)	0.0034 (7)	0.0018 (6)
O2	0.0272 (7)	0.0232 (8)	0.0249 (7)	0.0007 (7)	0.0030 (6)	0.0045 (6)
O3	0.0317 (8)	0.0247 (8)	0.0242 (7)	0.0083 (7)	0.0038 (6)	−0.0033 (6)
O4	0.0346 (9)	0.0363 (11)	0.0491 (10)	−0.0068 (8)	−0.0037 (8)	0.0064 (8)
O5	0.0261 (8)	0.0365 (10)	0.0328 (8)	0.0001 (7)	0.0049 (7)	0.0009 (7)
C1	0.0243 (11)	0.0257 (11)	0.0270 (11)	−0.0035 (9)	−0.0029 (9)	0.0000 (9)
C2	0.0296 (11)	0.0238 (12)	0.0191 (10)	0.0010 (9)	−0.0012 (8)	−0.0034 (8)
C3	0.0302 (11)	0.0268 (12)	0.0212 (10)	0.0021 (9)	−0.0011 (9)	−0.0029 (9)
C4	0.0310 (11)	0.0209 (11)	0.0228 (10)	0.0039 (9)	0.0008 (8)	0.0010 (8)
C5	0.0204 (10)	0.0278 (12)	0.0250 (11)	0.0053 (9)	0.0005 (8)	−0.0015 (9)
C6	0.0289 (11)	0.0261 (12)	0.0249 (10)	0.0067 (10)	0.0049 (8)	−0.0003 (9)
C7	0.0276 (10)	0.0206 (11)	0.0212 (9)	0.0013 (9)	0.0034 (8)	−0.0016 (8)
C8	0.0239 (10)	0.0185 (10)	0.0195 (9)	0.0008 (9)	0.0045 (8)	−0.0017 (8)
C9	0.0202 (9)	0.0204 (10)	0.0207 (9)	−0.0017 (8)	0.0025 (8)	−0.0025 (8)
C10	0.0218 (10)	0.0256 (12)	0.0217 (10)	0.0006 (9)	0.0027 (8)	−0.0006 (9)
C11	0.0248 (10)	0.0221 (11)	0.0208 (9)	−0.0031 (9)	−0.0003 (8)	0.0001 (8)
C12	0.0249 (10)	0.0175 (11)	0.0216 (9)	−0.0008 (9)	0.0015 (8)	0.0006 (8)
C13	0.0213 (10)	0.0188 (11)	0.0171 (9)	−0.0018 (8)	0.0034 (8)	0.0004 (8)
C14	0.0216 (10)	0.0163 (10)	0.0196 (9)	−0.0027 (8)	0.0048 (8)	0.0003 (8)
C15	0.0279 (10)	0.0219 (11)	0.0228 (10)	−0.0020 (9)	0.0049 (8)	−0.0035 (8)
C16	0.0251 (11)	0.0231 (11)	0.0246 (10)	−0.0043 (9)	0.0016 (8)	−0.0019 (9)
C17	0.0187 (10)	0.0212 (10)	0.0187 (9)	−0.0023 (8)	0.0055 (8)	−0.0015 (8)
C18	0.0220 (10)	0.0289 (12)	0.0241 (10)	−0.0022 (9)	0.0063 (8)	0.0036 (9)
C19	0.0232 (10)	0.0425 (14)	0.0339 (12)	−0.0033 (11)	0.0064 (9)	0.0007 (11)
C20	0.0222 (10)	0.0271 (11)	0.0176 (9)	−0.0004 (9)	0.0047 (8)	0.0018 (8)
C21	0.0306 (12)	0.0275 (12)	0.0248 (11)	0.0028 (10)	0.0012 (9)	0.0030 (9)
C22	0.0234 (10)	0.0311 (12)	0.0228 (10)	−0.0009 (9)	0.0027 (8)	0.0034 (9)
C23	0.0294 (12)	0.0474 (16)	0.0321 (11)	−0.0058 (12)	−0.0028 (9)	0.0134 (11)
C24	0.0309 (12)	0.0353 (14)	0.0209 (10)	−0.0039 (10)	0.0015 (9)	0.0003 (9)
N1A	0.114 (5)	0.067 (4)	0.072 (4)	0.017 (4)	0.033 (4)	0.023 (3)
C2A	0.099 (4)	0.080 (4)	0.053 (5)	0.049 (5)	0.007 (4)	0.002 (3)
C3A	0.052 (4)	0.064 (5)	0.047 (3)	0.006 (4)	0.009 (3)	0.002 (3)
N4A	0.060 (3)	0.062 (4)	0.048 (3)	0.003 (3)	−0.001 (2)	−0.008 (3)
C5A	0.099 (4)	0.080 (4)	0.053 (5)	0.049 (5)	0.007 (4)	0.002 (3)
C6A	0.073 (5)	0.063 (6)	0.081 (6)	0.021 (5)	−0.003 (5)	−0.027 (5)
C7A	0.051 (4)	0.055 (4)	0.066 (4)	−0.005 (3)	0.012 (3)	−0.024 (4)
C8A	0.064 (4)	0.064 (5)	0.071 (4)	−0.035 (4)	0.019 (3)	−0.007 (4)
C9A	0.046 (3)	0.045 (4)	0.070 (4)	−0.012 (3)	0.018 (3)	−0.013 (3)
C10A	0.032 (3)	0.050 (4)	0.058 (3)	−0.001 (3)	−0.004 (2)	−0.007 (3)

### Geometric parameters (Å, °)

**Table:**

O1—C3	1.449 (3)	C14—H14A	0.9800
O1—H1O	0.8200	C15—C16	1.535 (3)
O2—C7	1.438 (2)	C15—H15A	0.9700
O2—H2O	0.8200	C15—H15B	0.9700
O3—C12	1.438 (2)	C16—C17	1.564 (3)
O3—H3O	0.8200	C16—H16A	0.9700
O4—C24	1.213 (3)	C16—H16B	0.9700
O5—C24	1.317 (3)	C17—C20	1.539 (3)
O5—H5O	0.8200	C17—H17A	0.9800
C1—C2	1.523 (3)	C18—H18A	0.9600
C1—C10	1.545 (3)	C18—H18B	0.9600
C1—H1A	0.9700	C18—H18C	0.9600
C1—H1B	0.9700	C19—H19A	0.9600
C2—C3	1.515 (3)	C19—H19B	0.9600
C2—H2A	0.9700	C19—H19C	0.9600
C2—H2B	0.9700	C20—C21	1.526 (3)
C3—C4	1.508 (3)	C20—C22	1.537 (3)
C3—H3A	0.9800	C20—H20A	0.9800
C4—C5	1.537 (3)	C21—H21A	0.9600
C4—H4A	0.9700	C21—H21B	0.9600
C4—H4B	0.9700	C21—H21C	0.9600
C5—C6	1.534 (3)	C22—C23	1.510 (3)
C5—C10	1.555 (3)	C22—H22A	0.9700
C5—H5A	0.9800	C22—H22B	0.9700
C6—C7	1.528 (3)	C23—C24	1.512 (3)
C6—H6A	0.9700	C23—H23A	0.9700
C6—H6B	0.9700	C23—H23B	0.9700
C7—C8	1.532 (3)	N1A—C2A	1.332 (15)
C7—H7A	0.9800	N1A—C9A	1.366 (10)
C8—C14	1.519 (3)	C2A—C3A	1.440 (19)
C8—C9	1.547 (3)	C2A—H2Q	0.9600
C8—H8A	0.9800	C3A—N4A	1.294 (11)
C9—C11	1.541 (3)	C3A—H3Q	0.9600
C9—C10	1.564 (3)	N4A—C10A	1.329 (8)
C9—H9A	0.9800	C5A—C6A	1.292 (18)
C10—C19	1.540 (3)	C5A—C10A	1.503 (13)
C11—C12	1.534 (3)	C5A—H5Q	0.9600
C11—H11A	0.9700	C6A—C7A	1.346 (13)
C11—H11B	0.9700	C6A—H6Q	0.9600
C12—C13	1.552 (3)	C7A—C8A	1.327 (11)
C12—H12A	0.9800	C7A—H7Q	0.9600
C13—C18	1.534 (3)	C8A—C9A	1.424 (10)
C13—C14	1.541 (3)	C8A—H8Q	0.9600
C13—C17	1.557 (3)	C9A—C10A	1.397 (10)
C14—C15	1.527 (3)		
			
C3—O1—H1O	109.5	C15—C14—H14A	106.3
C7—O2—H2O	109.5	C13—C14—H14A	106.3
C12—O3—H3O	109.5	C14—C15—C16	103.41 (17)
C24—O5—H5O	109.5	C14—C15—H15A	111.0
C2—C1—C10	114.76 (18)	C16—C15—H15A	111.2
C2—C1—H1A	108.6	C14—C15—H15B	111.0
C10—C1—H1A	108.5	C16—C15—H15B	111.1
C2—C1—H1B	108.5	H15A—C15—H15B	109.1
C10—C1—H1B	108.7	C15—C16—C17	107.41 (17)
H1A—C1—H1B	107.5	C15—C16—H16A	110.2
C3—C2—C1	110.47 (18)	C17—C16—H16A	110.2
C3—C2—H2A	109.5	C15—C16—H16B	110.2
C1—C2—H2A	109.5	C17—C16—H16B	110.2
C3—C2—H2B	109.6	H16A—C16—H16B	108.5
C1—C2—H2B	109.6	C20—C17—C13	119.94 (16)
H2A—C2—H2B	108.1	C20—C17—C16	111.41 (16)
O1—C3—C4	108.80 (18)	C13—C17—C16	103.25 (16)
O1—C3—C2	109.21 (18)	C20—C17—H17A	107.2
C4—C3—C2	109.87 (16)	C13—C17—H17A	107.2
O1—C3—H3A	109.6	C16—C17—H17A	107.2
C4—C3—H3A	109.6	C13—C18—H18A	109.5
C2—C3—H3A	109.7	C13—C18—H18B	109.5
C3—C4—C5	113.84 (18)	H18A—C18—H18B	109.5
C3—C4—H4A	108.8	C13—C18—H18C	109.5
C5—C4—H4A	108.8	H18A—C18—H18C	109.5
C3—C4—H4B	108.8	H18B—C18—H18C	109.5
C5—C4—H4B	108.8	C10—C19—H19A	109.5
H4A—C4—H4B	107.7	C10—C19—H19B	109.5
C6—C5—C4	109.95 (18)	H19A—C19—H19B	109.5
C6—C5—C10	112.59 (17)	C10—C19—H19C	109.5
C4—C5—C10	113.20 (17)	H19A—C19—H19C	109.5
C6—C5—H5A	106.8	H19B—C19—H19C	109.5
C4—C5—H5A	107.0	C21—C20—C22	110.95 (19)
C10—C5—H5A	106.9	C21—C20—C17	113.38 (16)
C7—C6—C5	114.47 (18)	C22—C20—C17	107.83 (17)
C7—C6—H6A	108.6	C21—C20—H20A	108.2
C5—C6—H6A	108.7	C22—C20—H20A	108.1
C7—C6—H6B	108.7	C17—C20—H20A	108.2
C5—C6—H6B	108.7	C20—C21—H21A	109.5
H6A—C6—H6B	107.6	C20—C21—H21B	109.5
O2—C7—C6	112.56 (16)	H21A—C21—H21B	109.5
O2—C7—C8	107.32 (16)	C20—C21—H21C	109.5
C6—C7—C8	111.10 (18)	H21A—C21—H21C	109.5
O2—C7—H7A	108.6	H21B—C21—H21C	109.5
C6—C7—H7A	108.6	C23—C22—C20	115.66 (19)
C8—C7—H7A	108.6	C23—C22—H22A	108.4
C14—C8—C7	111.34 (17)	C20—C22—H22A	108.4
C14—C8—C9	109.29 (16)	C23—C22—H22B	108.4
C7—C8—C9	112.75 (16)	C20—C22—H22B	108.3
C14—C8—H8A	107.8	H22A—C22—H22B	107.4
C7—C8—H8A	107.7	C22—C23—C24	111.6 (2)
C9—C8—H8A	107.7	C22—C23—H23A	109.3
C11—C9—C8	109.61 (16)	C24—C23—H23A	109.3
C11—C9—C10	113.74 (17)	C22—C23—H23B	109.3
C8—C9—C10	111.89 (16)	C24—C23—H23B	109.3
C11—C9—H9A	107.1	H23A—C23—H23B	108.0
C8—C9—H9A	107.1	O4—C24—O5	123.7 (2)
C10—C9—H9A	107.0	O4—C24—C23	123.4 (2)
C19—C10—C1	106.71 (18)	O5—C24—C23	112.9 (2)
C19—C10—C5	109.08 (19)	C2A—N1A—C9A	117.3 (9)
C1—C10—C5	107.73 (16)	N1A—C2A—C3A	119.3 (10)
C19—C10—C9	111.32 (17)	N1A—C2A—H2Q	120.8
C1—C10—C9	112.12 (18)	C3A—C2A—H2Q	118.6
C5—C10—C9	109.75 (17)	N4A—C3A—C2A	123.7 (8)
C12—C11—C9	114.72 (17)	N4A—C3A—H3Q	116.9
C12—C11—H11A	108.5	C2A—C3A—H3Q	118.9
C9—C11—H11A	108.6	C3A—N4A—C10A	116.4 (6)
C12—C11—H11B	108.7	C6A—C5A—C10A	119.7 (10)
C9—C11—H11B	108.5	C6A—C5A—H5Q	120.0
H11A—C11—H11B	107.6	C10A—C5A—H5Q	120.2
O3—C12—C11	107.76 (15)	C5A—C6A—C7A	123.0 (8)
O3—C12—C13	111.10 (16)	C5A—C6A—H6Q	121.9
C11—C12—C13	110.99 (17)	C7A—C6A—H6Q	115.1
O3—C12—H12A	109.0	C8A—C7A—C6A	121.6 (7)
C11—C12—H12A	109.0	C8A—C7A—H7Q	118.4
C13—C12—H12A	108.9	C6A—C7A—H7Q	120.0
C18—C13—C14	112.59 (17)	C7A—C8A—C9A	120.6 (7)
C18—C13—C12	109.39 (16)	C7A—C8A—H8Q	120.0
C14—C13—C12	106.79 (15)	C9A—C8A—H8Q	119.4
C18—C13—C17	109.80 (15)	N1A—C9A—C10A	120.4 (6)
C14—C13—C17	99.98 (16)	N1A—C9A—C8A	120.6 (7)
C12—C13—C17	118.02 (17)	C10A—C9A—C8A	118.9 (7)
C8—C14—C15	117.58 (17)	N4A—C10A—C9A	122.9 (6)
C8—C14—C13	115.05 (17)	N4A—C10A—C5A	121.1 (8)
C15—C14—C13	104.49 (15)	C9A—C10A—C5A	116.0 (8)
C8—C14—H14A	106.4		
			
C10—C1—C2—C3	58.4 (2)	C7—C8—C14—C13	−175.02 (16)
C1—C2—C3—O1	−175.59 (17)	C9—C8—C14—C13	59.8 (2)
C1—C2—C3—C4	−56.3 (2)	C18—C13—C14—C8	60.7 (2)
O1—C3—C4—C5	174.60 (17)	C12—C13—C14—C8	−59.4 (2)
C2—C3—C4—C5	55.1 (2)	C17—C13—C14—C8	177.19 (16)
C3—C4—C5—C6	−179.90 (18)	C18—C13—C14—C15	−69.7 (2)
C3—C4—C5—C10	−53.0 (2)	C12—C13—C14—C15	170.20 (16)
C4—C5—C6—C7	75.1 (2)	C17—C13—C14—C15	46.75 (18)
C10—C5—C6—C7	−52.1 (2)	C8—C14—C15—C16	−164.70 (17)
C5—C6—C7—O2	−69.4 (2)	C13—C14—C15—C16	−35.8 (2)
C5—C6—C7—C8	51.0 (2)	C14—C15—C16—C17	10.5 (2)
O2—C7—C8—C14	−52.2 (2)	C18—C13—C17—C20	−44.7 (2)
C6—C7—C8—C14	−175.64 (16)	C14—C13—C17—C20	−163.28 (17)
O2—C7—C8—C9	71.1 (2)	C12—C13—C17—C20	81.5 (2)
C6—C7—C8—C9	−52.4 (2)	C18—C13—C17—C16	79.92 (19)
C14—C8—C9—C11	−53.0 (2)	C14—C13—C17—C16	−38.63 (17)
C7—C8—C9—C11	−177.41 (17)	C12—C13—C17—C16	−153.84 (17)
C14—C8—C9—C10	179.85 (17)	C15—C16—C17—C20	147.96 (16)
C7—C8—C9—C10	55.4 (2)	C15—C16—C17—C13	17.9 (2)
C2—C1—C10—C19	−170.19 (19)	C13—C17—C20—C21	−57.8 (2)
C2—C1—C10—C5	−53.2 (2)	C16—C17—C20—C21	−178.47 (17)
C2—C1—C10—C9	67.7 (2)	C13—C17—C20—C22	178.95 (18)
C6—C5—C10—C19	−69.9 (2)	C16—C17—C20—C22	58.3 (2)
C4—C5—C10—C19	164.58 (17)	C21—C20—C22—C23	65.8 (3)
C6—C5—C10—C1	174.60 (17)	C17—C20—C22—C23	−169.5 (2)
C4—C5—C10—C1	49.1 (2)	C20—C22—C23—C24	175.6 (2)
C6—C5—C10—C9	52.3 (2)	C22—C23—C24—O4	−31.5 (4)
C4—C5—C10—C9	−73.2 (2)	C22—C23—C24—O5	147.5 (2)
C11—C9—C10—C19	−58.2 (2)	C9A—N1A—C2A—C3A	−2.5 (18)
C8—C9—C10—C19	66.7 (2)	N1A—C2A—C3A—N4A	4.0 (18)
C11—C9—C10—C1	61.3 (2)	C2A—C3A—N4A—C10A	−2.8 (12)
C8—C9—C10—C1	−173.85 (16)	C10A—C5A—C6A—C7A	0(2)
C11—C9—C10—C5	−179.05 (16)	C5A—C6A—C7A—C8A	−2.9 (16)
C8—C9—C10—C5	−54.2 (2)	C6A—C7A—C8A—C9A	2.5 (10)
C8—C9—C11—C12	53.5 (2)	C2A—N1A—C9A—C10A	0.2 (13)
C10—C9—C11—C12	179.60 (16)	C2A—N1A—C9A—C8A	−175.9 (10)
C9—C11—C12—O3	66.7 (2)	C7A—C8A—C9A—N1A	177.3 (7)
C9—C11—C12—C13	−55.2 (2)	C7A—C8A—C9A—C10A	1.1 (9)
O3—C12—C13—C18	172.15 (16)	C3A—N4A—C10A—C9A	0.3 (9)
C11—C12—C13—C18	−68.0 (2)	C3A—N4A—C10A—C5A	−178.4 (10)
O3—C12—C13—C14	−65.74 (19)	N1A—C9A—C10A—N4A	1.0 (10)
C11—C12—C13—C14	54.1 (2)	C8A—C9A—C10A—N4A	177.2 (6)
O3—C12—C13—C17	45.7 (2)	N1A—C9A—C10A—C5A	179.8 (10)
C11—C12—C13—C17	165.59 (16)	C8A—C9A—C10A—C5A	−4.0 (11)
C7—C8—C14—C15	−51.3 (2)	C6A—C5A—C10A—N4A	−177.4 (11)
C9—C8—C14—C15	−176.48 (16)	C6A—C5A—C10A—C9A	3.8 (18)

### Hydrogen-bond geometry (Å, °)

**Table:**

*D*—H···*A*	*D*—H	H···*A*	*D*···*A*	*D*—H···*A*
O1—H1O···O3^i^	0.82	2.03	2.815 (2)	161
O2—H2O···O1^i^	0.82	1.86	2.648 (2)	160
O3—H3O···O4^ii^	0.82	2.03	2.834 (2)	167
O5—H5O···O2^ii^	0.82	1.85	2.656 (2)	170

Symmetry codes: (i) −*x*+1, *y*−1/2, −*z*+2; (ii) −*x*, *y*+1/2, −*z*+1.
